# Genome‐resolved metagenomics identifies novel active microbes in biogeochemical cycling within methanol‐enriched soil

**DOI:** 10.1111/1758-2229.13246

**Published:** 2024-04-04

**Authors:** Michael C. Macey

**Affiliations:** ^1^ AstrobiologyOU, Earth, Environment and Ecosystem Sciences The Open University Milton Keynes UK

## Abstract

Metagenome assembled genomes (MAGs), generated from sequenced ^13^C‐labelled DNA from ^13^C‐methanol enriched soils, were binned using an ensemble approach. This method produced a significantly larger number of higher‐quality MAGs compared to direct binning approaches. These MAGs represent both the primary methanol utilizers and the secondary utilizers labelled via cross‐feeding and predation on the labelled methylotrophs, including numerous uncultivated taxa. Analysis of these MAGs enabled the identification of multiple metabolic pathways within these active taxa that have climatic relevance relating to nitrogen, sulfur and trace gas metabolism. This includes denitrification, dissimilatory nitrate reduction to ammonium, ammonia oxidation and metabolism of organic sulfur species. The binning of viral sequence data also yielded extensive viral MAGs, identifying active viral replication by both lytic and lysogenic phages within the methanol‐enriched soils. These MAGs represent a valuable resource for characterizing biogeochemical cycling within terrestrial environments.

## Graphical abstract

Ensemble binning of environmental metagenomes significantly improves the analysis of complex datasets. A re‐analysis of metagenomes, derived from labelled DNA extracted from ^13^C methanol‐enriched rhizosphere soils, yielded a substantial number of metagenome‐assembled genomes. This facilitated the identification of numerous biogeochemical cycling pathways, including those relevant to climate change. The binning process further revealed metagenome‐assembled genomes (MAGs) of bacterial predators and viruses, thereby uncovering trophic interactions within the enriched soils. This dataset serves as a valuable resource for characterising biogeochemical cycling within terrestrial environments.

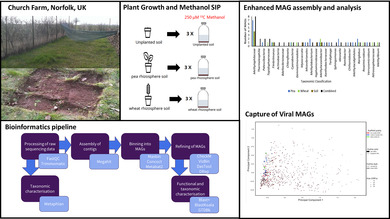

## INTRODUCTION

Soils play a major role in global biogeochemical cycling, including the synthesis and metabolism of gases relevant to climate change (e.g., methane, nitrous oxide, methanol, carbon monoxide). Amongst these volatiles, methanol represents the third most emitted volatile organic gas (120–350 Tg yr^−1^), after methane (535 Tg yr^−1^) and isoprene (350–520 Tg yr^−1^) (Opacka et al., 2021). Methanol and its products from atmospheric interaction will act as either a net source or a net sink for radicals, which can in turn influence the lifetime of methane in the atmosphere (Galbally & Kirstine, 2002; Heikes et al., 2002). Methanol can be converted to formic acid by photochemical reactions that can enhance the formation of acid rain (Jacob, 1986).

Methanol‐utilising methylotrophs can also play a major role in the cycling of further climate‐impacting compounds with relevance to global biogeochemical cycling of the major elements (e.g., nitrous oxide production and consumption *via* denitrification [Abed et al., 2013; Prosser et al., 2020; Wrage et al., 2004], production and oxidation of dimethyl‐sulfide [DMS] [Carrión et al., 2017; Zhang et al., 2019]). Characterisation of the role of this functional guild of microbes in biogeochemical cycling in soils requires the identification of their genomic potential within the soil microbiome. Stable isotope probing (SIP) is a powerful technique that can identify active substrate utilizers in an environment whilst reducing the issues with sequencing depth associated with complex microbial communities and identification of the specific microbes involved in a defined metabolic process (Chen & Murrell, 2010; Radajewski et al., 2002; Thomas et al., 2019). Incubation length is a key component of the design of SIP experiments—identification of environmentally relevant microbes requires incubations with environmentally relevant substrate concentrations in the absence of further nutritional amendments, as these can influence the microbial community (Cébron et al., 2007; Chen et al., 2008; McDonald et al., 2005); however, this can result in long‐term incubations, which in turn enhances the ability of non‐target microbes to incorporate the ^13^C label into their biomass *via* cross‐feeding (Lueders et al., 2006; Macey et al., 2020). However, in addition to the generation of a focussed metagenome enabling the identification of the taxonomic and functional potential of the microbial community, genome‐resolved metagenomics can inform the identification of primary and secondary ^13^C utilizers.

In a previous study, we performed a DNA‐SIP experiment with ^13^C‐labelled methanol to identify methylotrophs in pea and wheat rhizosphere soil relative to the unplanted soil to identify the impact of plant growth on the soil community (Macey et al., 2020). This study identified that *Methylobacterium* was enriched in the rhizosphere soils relative to the unplanted soils, potentially due to exudates and plant‐generated methanol (Galbally & Kirstine, 2002). This potential for enrichment of soil methylotrophs in association with plants has also been observed *via* other studies that performed SIP experiments with *Zea mays*, *Arabidopsis thaliana*, grassland and deciduous forest soils (Butterfield et al., 2016; Chaignaud et al., 2018; Li et al., 2019; Morawe et al., 2017). The metagenomes generated from the ^13^C labelled DNA from the previous Macey et al., 2020 study were analysed with a direct binning approach. This enabled the identification of 10 metagenome‐assembled genomes (MAGs), which included the most abundant methanol utilizers across four MAGs and six additional MAGs that represented potential cross‐feeders. However, *via* an ensemble approach, it is possible to identify a greater number of MAGs (Yue et al., 2020) and detect MAGs representative of a greater diversity of taxa in the active soil community. Across multiple studies, direct binning programs are shown to vary in their relative performance across different environments and sample types (Bak et al., 2023; Wang et al., 2023) (e.g., variable numbers of bins, completion, and contamination). Ensemble binning methods allow the integration and dereplication of binning results of multiple direct binning programs—an ensemble binning approach can therefore generate a higher number of good quality MAGs without the high levels of redundancy from directly utilising the outputs of direct binning programs.

In this study, we applied an ensemble approach to enhance the identification of the taxonomic and functional potential of the active carbon cycling component of the soil microbiome following enrichment with methanol. Viral MAGs were also assembled and characterised, as these have previously been shown to have a major impact on both enriched diversity and carbon cycling (Barnett & Buckley, 2023; Starr et al., 2021; Trubl et al., 2021).

## EXPERIMENTAL PROCEDURES

### Sample description and sequencing

In the originating study (Macey et al., 2020), the soil was collected in April 2015 from a naturally grassed and unfertilised part of John Innes Centre Church Farm (Norfolk, UK) (52.6276 N, 1.1786 E). The top 10 cm was removed from a 1 m^2^ section and then soil to 20 cm depth was removed, air dried and sieved through 10 mm and 5 mm sieves. Sterilised *Pisum sativa* and *Triticum aestivum* seeds were then planted and grown in 10 cm × 10 cm pots in bulk soil and growing at 22°C under long day regimes (16:8 h) in plant growth rooms for 4 weeks. Unplanted controls were run alongside. Rhizosphere soil was collected by shaking the plant roots three times to separate non‐rhizosphere soil (Bulgarelli et al., 2012). Roots were then vortexed in autoclaved 1× phosphate buffered saline (PBS) (In 1 L of water: NaCl 8 g, KCl 0.2 g, Na_2_HPO_4_ 1.44 g and KH_2_PO_4_ 0.24 g) to separate the adhering soil that is defined as rhizosphere soil. 2 g of rhizosphere soil and unplanted soil was dispensed into 120‐ml serum vials in triplicate and combined with 40 mL of sterile H_2_O (soil slurry). Vials were then supplemented with 10 μmol of ^13^C methanol or ^12^C methanol. The serum vials were sealed air‐tight with rubber seals and then incubated at 30°C without light in a shaking incubator (120 rpm). These vials were incubated alongside uninoculated vials with 40 mL of sterile H_2_O incubated with 100 μM–1 mM methanol to act as standards for the concentration of methanol. The concentration of methanol in the headspace of the serum vials was measured using gas chromatography as detailed in Macey et al., 2020. After depletion of methanol, samples were resupplied with methanol to the same concentration. After 17 days, a total of 200 μmol of C had been consumed in incubations and soil was collected for DNA extraction. DNA was extracted from all soil samples via the Griffiths technique (Griffiths et al., 2000). DNA was processed via caesium chloride density gradient centrifugation to separate the ^13^C‐ and ^12^C‐labelled DNA from 1 to 3 μg of DNA from each test group according to established protocols and DNA was recovered by precipitation (Neufeld et al., 2007). Confirmation of ^13^C labelling was achieved *via* denaturing gradient gel electrophoresis and 16S rRNA gene amplicon sequencing of the heavy and light fractions of the DNA extracted from the ^12^C and ^13^C enriched test groups to confirm the occurrence of enriched taxa, as detailed in Taubert et al., 2015, Taubert et al., 2019 and Macey et al., 2020. The DNA from the ^13^C‐heavy fractions of the methanol SIP experiment was then pooled, quantified, and sequenced by the Centre for Genomic Research at the University of Liverpool. Sequencing was performed using paired‐end sequencing (2 × 150 bp) on an Illumina HiSeq 4000.

### Metagenome analysis and assembly of metagenome assembled genomes

Short sequences and sequences of poor quality were excluded from the files using the program Trimmomatic (0.36) (Bolger et al., 2014) and the quality of the metagenomes was assessed using QUAST, including the MetaQUAST expansion (5.0.0) (Gurevich et al., 2013). The reads from the pea, wheat and unplanted soil methanol enrichments were pooled to enhance the representation of less abundant taxa and these were processed alongside the individual metagenomes. Reads were assembled using Megahit (1.1.2) (Li et al., 2015). The Trimmomatic and Megahit steps were performed using Kbase (Arkin et al., 2018). The taxonomic profile of the metagenome was analysed using Metaphlan3 (3.0) (Beghini et al., 2021; Segata et al., 2013).

Contigs were binned into metagenome‐assembled genomes (MAGs) using the binning programs MaxBin2 (2.2.4) (Wu et al., 2016) (version), Metabat2 (1.7) (Kang et al., 2019) and Concoct (1.1) (Alneberg et al., 2014). The MAGs were then processed into a non‐redundant set of MAGs using the program DasTool (1.1.2) (Sieber et al., 2018) and dRep (3.1) (Olm et al., 2017) with default settings. The completeness, contamination and heterogeneity of these MAGs were assessed using the program CheckM (1.0.13) (Parks et al., 2015). MAGs within 10% of a contamination quality score threshold (5% for high quality and 10% for medium quality) were refined using VizBin (Laczny et al., 2015). The taxonomy of the MAGs was then assessed using GTDBk and MAGs with taxonomic identification above the family level were further assessed using TYGS and Protologger (Chaumeil et al., 2020, 2022; Hitch et al., 2021; Meier‐Kolthoff & Göker, 2019). The presence of specific functional genes, metabolic pathways, rRNAs and tRNAs was also screened for using BlastKOALA (Kanehisa et al., 2016) and DRAM (Shaffer et al., 2020). Genes of interest were also screened for by performing local BLAST searches in BioEdit with tblastn against local databases generated from the MAGs (Hall, 1999). The abundance of the MAGs in the reads of the metagenomes was analysed using Bowtie2 (Langmead & Salzberg, 2012). Viral sequences were recovered, binned and annotated from the metagenomes using the program VIBRANT (Kieft et al., 2020). The taxonomy of the viral sequences was then assessed using the program VirSorter (Guo et al., 2021).

## RESULTS AND DISCUSSION

### Ensemble and direct binning approaches

Ensemble binning of the MAGs from the metagenomes generated from the methanol‐enriched soils generated 23 MAGs from the pea rhizosphere soil, 14 MAGs from the wheat rhizosphere soil and 8 MAGs from the unplanted soil (Supplementary Figure 1). The MAGs generated from the rhizosphere soils covered a greater taxonomic diversity. MAGs identified as *Cyanobacteria* and *Rariglobus* were both identified exclusively in the rhizosphere soils, as well as additional MAGs identified as *Methylophilaceae* (seven as opposed to four). Further MAGs exclusive to the methanol‐enriched Pea rhizosphere soil were identified as *Patescibacteria, Methylobacterium, Xanthobacteraceae, Tardiphaga, Ideonella, Chthoniobacter, Methylacidiphilales* and *Verrucomicrobium*. The methanol‐enriched wheat rhizosphere soils exclusively contained MAGs identified as *Gemmatimonadales*, *Roseimicrobium* and *Nitrososphaeraceae*. This supports the prior identification of a greater diversity of these specific taxa in the methanol‐enriched rhizosphere soils relative to the unplanted soils (Macey et al., 2020), with many of the taxa previously identified as enriched in rhizosphere soils (e.g., *Chthoniobacter* [Sangwan et al., 2004], *Nitrososphaeraceae* [Prudence et al., 2021], *Methylophilaceae* [Kanukollu et al., 2022; Macey et al., 2020], and species within the Acidobacteriota and Verrucomicrobiota [Da Rocha et al., 2013; Lanzavecchia et al., 2024]).

Ensemble binning of MAGs from the combined methanol‐enriched soil metagenomes generated 48 microbial MAGs of medium quality (>50% completeness and <10% contamination) (Table 1). For MAGs of this quality threshold, Concoct generated 25, Metabat 22 and Maxbin 21. DRep did not identify any MAGs as redundant, supporting that the binning programs generated partially distinct sets of MAGs. Prior analysis of the metagenomes (Macey et al., 2020) generated 18 MAGs of at least medium quality, 11 Pseudomonodata MAGs, with additional detection of five Verrucomicrobiota and one Bdellovibrionota and Thermoproteota MAGs. Pseudomonodata and Verrucomicrobiota were also the most abundant phyla in the MAGs generated in this study, with 18 and five MAGs respectively. One Thermoproteota and one Bdellovibrionota MAG were also generated. This study also generated MAGs from eight further phyla: Actinobacteriota (3), Planctomycetota (2), Patescibacteria (2), Myxococcota (1), Gemmatimonadota (1), Cyanobacteriota (1), Chloroflexota (1), Armatimonadota (1). Bins 5, 12, 19, 30 and 43 all had contamination scores above 10%, identifying them as low‐quality MAGs. However, *via* manual selection of the MAG contigs with VizBin, it was possible to reduce their contamination levels to below 10%, increasing the quality assignment of these MAGs. MAG completeness remained above 50% in all instances. The MAGs included those that could not be assigned to high levels of taxonomic resolution, being classified to the levels of Family (4 MAGs – *Hyphomicrobiaceae, Methylophilaceae, Bdellovibrionaceae, Nitrososphaeraceae*), Order (2 MAGs – Gemmatimonadales, Micavibrionales), Class (3 MAGs Thermoleophilia, Physciphaerae, Sericytochromatia) and Phylum (6 MAGs – Planctomycetota, Myxococcota, Actinobacteriota, Chloroflexota and Patescibacteria). These MAGs are of value for characterising members of these uncultivated taxa, including attempting to elucidate their potential role in biogeochemical cycling in the soil environment. Collectively, the MAGs represent 25.77% of the mapped reads, with 11.18% of the mapped reads represented by bin 46, a *Methylophilus* MAG. Of the five MAGs that represented >1% of the mapped reads, three were also identified as *Methylophilaceae* (bins 1, 7 and 26). The fourth bin was identified as bin 30, which contained *xoxF* and was identified as *Ramlibacter*. The fifth bin representing >1% of the mapped reads were identified as *Rariglobus* (previously identified as *Opitutus* in Macey et al., 2020), which was identified as a 1% relative abundance of the enriched microbiome.

**TABLE 1:** Taxonomic classification, quality score and RNA contents of the metagenome assembled genomes.

	Classification by GTDBk	Contigs	Completeness %	Contamination%	5S rRNA	16S rRNA	23S rRNA	tRNA count	Abundance in reads^a^	Relative abundance in community profile^a^
bin.001.fa	*Methylophilus*	259	68.22	7.51				21	1.2	20
bin.002.fa	*Methylibium*	100	91.84	9.84				41	0.16	0.2
bin.003.fa	*Methylobacterium*	137	86.29	5.22	2			37	0.28	2
bin.004.fa	*Hyphomicrobiaceae* AWTP1‐13	463	94.49	5.11				57	0.24	4
bin.005.fa	*Sericytochromatia* UBA7694	1726	77.26	6.41				74	0.26	0.4
bin.006.fa	*Roseimicrobium*	833	56.08	3.43				18	0.14	0.9
bin.007.fa	*Methylophilaceae* MM2	118	93.76	9.93				42	1.8	29
bin.008.fa	*Fimbriimonas*	268	91.51	1.47				47	0.09	0
bin.009.fa	*Myxococcota* XYA12‐FULL‐58‐9	217	93.31	2.26		1		46	0.17	0.7
bin.012.fa	*Methylophilaceae*	403	82.89	7.95	1			40	0.94	29
bin.013.fa	*Methylophilaceae* MM1	137	94.02	6.92	2			36	0.39	29
bin.014.fa	*Bdellovibrionaceae* 21‐14‐0‐10‐47‐8	249	86.74	1.8				28	0.06	0.06
bin.016.fa	*Solirubrobacteraceae*	580	66.87	2.14				35	0.14	0.7
bin.017.fa	Thermoleophilia Ga0077560	509	70.52	1.03				34	0.08	0.7
bin.019.fa	Gemmatimonadales GWC2‐71‐9	932	58	9.6				33	0.09	0.2
bin.020.fa	*Verrucomicrobium*	1154	80.04	1.39				25	0.1	1
bin.021.fa	Patescibacteria UBA1384	69	62.74	1.03	1	1	1	38	0.03	0
bin.022.fa	*Methyloceanibacter*	510	90.78	7.8	1		1	47	0.8	1
bin.023.fa	*Phycisphaerae* UBA1161	813	86.33	5.75	2			49	0.21	2
bin.024.fa	*Methylophilaceae* MM2	278	86.26	8.3	1			48	0.73	29
bin.025.fa	Micavibrionales UBA2020	136	94.66	6.45				38	0.09	0.03
bin.026.fa	*Methylophilaceae* MM2	302	78.39	5.28	1			31	1.38	29
bin.027.fa	*Nitrososphaeraceae* TA‐21	160	96.15	0.97	1			37	0.05	0.06
bin.028.fa	*Methylophilaceae* MM1	30	86.7	0.06	2			29	0.14	29
bin.029.fa	*Chthoniobacter*	553	87.05	3.04				46	0.13	0
bin.030.fa	*Ramlibacter*	527	65	9.85	1			28	1.88	2
bin.031.fa	Planctomycetota J058	993	57.94	0.72				41	0.07	2
bin.032.fa	*Sphingomonas*	599	68.13	3.73	1			29	0.08	0.9
bin.033.fa	Micavibrionales UBA2020	256	87.6	8.97				45	0.2	0.03
bin.034.fa	Methylacidiphilales UBA1321	571	96.96	5.41				50	0.17	0.01
bin.035.fa	Paceibacteria UBA9983	139	39.45	1.79		2	1	26	0.02	0
bin.036.fa	Actinobacteriota UBA4738	206	89.74	5.3	1			48	0.08	7
bin.038.fa	*Ideonella*	75	97.2	2.25				54	0.47	0.2
bin.042.fa	*Xanthobacteraceae*	216	89.66	3.01	1			43	0.41	3
bin.043.fa	Chloroflexota Ellin6529	431	66.08	9.93				24	0.05	0.06
bin.044.fa	*Rariglobus*	359	84.13	2.4		1		45	1.46	1
bin.046.fa	*Methylophilus*	222	73.13	1.85				19	11.18	20
bin.047.fa	*Tardiphaga*	678	65.96	1.32	1			34	0.16	0.5

^a^ Abundance was calculated using Bowtie2 to compare the metagenome assembled genome (MAG) against the paired reads of the metagenomes. Relative abundance was derived from the abundance of the taxa most closely related to the MAG in the community profile.

### Identification of methylotrophic and non‐methylotrophic diversity

The prior analysis identified ten Pseudomonodata MAGs that contained genes encoding the *xoxF* methanol dehydrogenases and associated accessory genes (e.g., genes encoding for associated cytochromes or the mxaJ, a Periplasmic solute binding protein [Schmidt et al., 2010; Choi et al., 2013]) in the *Methylophilales* (8), *Methylobacterium* (1), and *Ramlibacter* (1) (Macey et al., 2020). Ensemble binning approaches applied in this study identified an additional five MAGS that either contained the *xoxF* methanol dehydrogenase gene and were assigned to taxa associated with methanol oxidation (*Xanthobacteraceae* [Tikhonova et al., 2021], *Ideonella* [Hachisuka et al., 2022] and *Methylibium* [Nakatsu et al., 2006; Song & Cho, 2007]) or were only assigned to taxa associated with methanol oxidation (*Methylacidiphilum* [Hou et al., 2008; Versantvoort et al., 2019] and *Methyloceanibacter* [Takeuchi et al., 2014, 2019]); whilst none of the 13 species of *Ramlibacter* is capable of methanol oxidation, members of other genera within the *Comamonadaceae* contain species confirmed to grow on methanol (Agafonova et al., 2017; Satola et al., 2013) and *xoxF* genes are present in members of 28 of the 34 genera of the family (Macey et al., 2020). As methanol oxidation is consistently tested in the absence of lanthanides, and these taxa only possess a lanthanide‐dependant methanol dehydrogenase (Fitriyanto et al., 2011; Hibi et al., 2011; Nakagawa et al., 2012; Wang et al., 2019), this metabolic trait may be undetected. Surprisingly, *mxaFI*, the canonical, non‐lanthanide dependant methanol dehydrogenase encoding genes were found in only three of the MAGs assembled in this study—bins 3 (*Methylobacterium*), 28 (*Methylophilaceae*) and 42 (*Xanthomonadaceae*).

Identification of the greater diversity of MAGs that were assigned to taxa not associated with methanol oxidation and that do not possess methanol dehydrogenases indicates that these additional MAGs could represent cross‐feeding microbes. The presence of these MAGs in the metagenome indicates that they were ^13^C labelled *via* utilisation of substrates generated by the methylotrophs either as a direct consequence of methanol oxidation by‐products from methanol oxidation (e.g., formaldehyde, formate, carbon dioxide), alternate substrates (sugars or exudates [Beck et al., 2013; Hernandez et al., 2015; Krause et al., 2017]) and/or labelled cellular material *via* necrophagy or predation (Barnett et al., 2016; Barnett & Buckley, 2023; Hanajima et al., 2019; López‐Mondéjar et al., 2020; Zumsteg et al., 2013). With regards to the utilisation of formaldehyde and formate, all the MAGs proposed as potential methylotrophs contained genes encoding for formaldehyde activating enzymes and formate—tetrahydrofolate ligases. These enzymes perform the condensation of formaldehyde and tetrahydromethanopterin to methylene tetrahydromethanopterin (Kim et al., 2020; Marx et al., 2003), which is then integrated into further formaldehyde and C1 metabolism pathways (Anthony, 1982.). A further 12 MAGs outside of the proposed methylotrophs contained formaldehyde‐activating enzymes and no further methylotrophy‐related genes, indicating a possible role in resolving issues associated with toxicity as opposed to metabolism (Vorholt et al., 2000; Yurimoto et al., 2005). Further to these genes, 20 complete formate dehydrogenase encoding genes were found across 10 MAGs; Bins 3, 4, 7 and 24 all contained two, and bins 12, 13, 26, 27, 28 and 38 contained one. Of these 10 MAGs, only bin 27, identified as *Nitrososphaeraceae*, was not a proposed methylotroph.

Prior SIP experiments have identified non‐methanotrophic methylotrophs and non‐methylotrophs labelled in ^13^C‐methane SIP experiments *via* the proposed metabolism of leaked labelled compounds (Beck et al., 2013; Taubert et al., 2019). Based on the shared metabolic pathway for complete methanol oxidation, these represent potential substrates in this experiment. The metabolic capabilities of the different groups indicate that they could have used many ^13^C compounds potentially produced by the methylotrophs. This includes carbon dioxide, compounds exuded by the methylotrophs or the cellular components of lysed methylotrophs (Pankratov et al., 2008; Noar & Buckley, 2009; Dumont et al., 2011). Necromass can represent a major organic carbon source in oligotrophic environments (Chatzigiannidou et al., 2018); therefore, it is also possible that some of the heterotrophic taxa incorporated the ^13^C label into their DNA *via* consumption of cellular material from dead labelled cells, as has previously been shown to occur in SIP experiments performed with ^13^C enriched dead *Eschericha coli* cells (Hanajima et al., 2019). These means of ^13^C labelling are well‐characterised issues with long‐term SIP experiments, indicating that the timescale of the methanol enrichment series was sufficient to allow this cross‐feeding to occur; this labelling also reinforces the balance between supplying an environmentally relevant concentration of a substrate, incorporating sufficient ^13^C to enable the collection of sufficient DNA for metagenome approaches and avoiding supplementation with additional nutrients to expedite the labelling of the cells. Furthermore, whilst not the primary utilizers of the labelled substrate, labelling that arises because of cross‐feeding does permit the identification and characterisation of active members of the soil microbial community. Bins 9 and 14 were assigned to bacterial taxa associated with predation of microbial cells (Myxococcota and *Bdellovibrionaceae* respectively). This labelling of predatory bacteria was previously observed in SIP experiments performed with ^13^C methane or ^13^C enriched prey cells and various terrestrial innocula (Barnett et al., 2016; Hutchens et al., 2004; Lueders et al., 2004). The methylotrophs enriched in these experiments were exclusively Gram‐negative, effectively generating an abundant source of labelled carbon for this specific metabolic guild of microbe.

### Biogeochemical cycling in the enriched soil

Multiple genes involved in the cycling of climatically relevant compounds were identified in the MAGs. Genes encoding for the complete dissimilatory nitrate reduction to ammonium (DNRA) (*narGHI* and *nirBD*) were detected in three MAGs, all non‐methylotrophic organisms (two Planctomycetota and one *Chthoniobacter*). As DNRA is typically associated with heterotrophy, this supports the identification of these labelled taxa as cross‐feeders. This metabolism is also associated with the conservation of nitrogen within an ecosystem (Pandey et al., 2020; Zhang et al., 2015)—these microbes may therefore play a role in the cycling of nitrogen within the soil environment as well as conserving the limited nitrogen within the batch culture approach of the enrichment. A MAG that was taxonomically assigned as an ammonia oxidising archaeon (AOA), being assigned to the *Nitrososphaeraceae*. This MAG contained a gene encoding for an ammonia monooxygenase, which performs the oxidation of ammonia to nitrite. As AOA have been shown to prefer environments with lower ammonia, this supports the low nutrient conditions of the enrichment series and the soil. It is also possible that there is an interchange of nitrogenous species between the AOA and DNRA. The enrichment of these functional groups also indicates that the enrichments may have transitioned to anoxic or microaerophilic conditions despite replenishment of the headspace (Macey et al., 2020). The enrichment of the AOA is also of note because they have been identified as inhibited by methanol concentrations that overlap with the supplied methanol concentration in this experiment (Macey et al., 2020; Oudova‐Rivera et al., 2023).

Three MAGs contained genes encoding the incomplete denitrification pathway—*Solirubrobacteraceae* missing the nitrous oxide reductase and *Hyphomicrobiaceae* and *Sphingomonas* only possessing the nitrate and nitrite reductase encoding genes, generating NO. Bins 24 and 26, taxonomically identified as *Methylophilaceae* (MM2) were the only MAGs that contained genes encoding for the complete denitrification pathway, as previously identified in the characterisation of the isolate *Methylobacillus* sp. MM2 (Macey et al., 2018). This is of note because N_2_O is the third most emitted greenhouse gas, with 300 times the forcing of CO_2_ and a 120‐year atmospheric lifespan, it is the most potent. Nitrous oxide is produced by microbes through either incomplete denitrification, converted from nitric oxide, or through the abiotic oxidation of hydroxylamine by ammonia‐oxidising microbes (Prosser et al., 2020). The production of N_2_O is also influenced by the other nitrogen cycling processes that increase the pool of nitrogenous species for denitrification (e.g., Annamox; Pajares & Ramos, 2019). Active microbes in this soil environment therefore appear to possess the genetic potential to have a major impact on inorganic and dissimilatory nitrogen metabolism.

With regards to carbon cycling, genes were identified that encoded the aerobic carbon monoxide dehydrogenase enzyme that catalyses the oxidation of carbon monoxide to carbon dioxide. Carbon Monoxide is strongly connected to methane and is considered “Kyoto's Forgotten Gas” (Fry et al., 2013), having been omitted from all current climate accords. CO oxidation‐related genes are broadly present in the microbiomes of terrestrial environments (Cordero et al., 2019) but the physiological process is poorly defined with regards to the ecology and physiology of CO‐oxidising microbes, with some organisms able to utilise this as either an energy source or a carbon and energy source. These genes were identified in four of the MAGs (*Thermoleophilia, Solirubrobacteraceae, Ramlibacter*, and *Hyphomicrobiaceae*); The genomes of isolated *Ramlibacter* species and *Solirubrobacteraceae* MAGs generated from DNA extracted from desert soils have both been shown to contain carbon monoxide dehydrogenase genes (Ray et al., 2022). Species within the *Hyphomicrobiaceae* have also been confirmed as capable of carbon monoxide oxidation (Volpiano et al., 2021). This observed genetic potential within taxonomically diverse and active soil microbes indicates a potential relevance of carboxydotrophy within this soil environment.

Further to the *cox* genes, the MAGs identified as *Solirubrobacteraceae* and *Hyphomicrobiaceae* also possessed the genes encoding for a methanethiol oxidase; this enzyme performs the oxidation of methanethiol to formaldehyde and hydrogen sulfide. Additional MAGs that contain this gene include the Gemmatimonadales, *Ideonella* and *Methylophilaceae*. Additional methanethiol metabolism‐related genes, encoding Methanethiol S Transferases, which catalyses the methylation of methanethiol (MeSH) to yield dimethylsulphide (DMS), were also found in multiple MAGs representative of both methylotrophic (*Hyphomicrobiaceae* and *Methylophilaceae*) and non‐methylotrophic taxa (*Verrucomicrobium, Planctomycetota* and *Tardiphaga*); members of the species of *Hyphomicrobiaceae* have been confirmed as capable of DMS oxidation (Pol et al., 1994) and *Methylophilaceae* have previously been indicated as active in terrestrial DMS degradation (Eyice et al., 2015), but the other identified taxa identified in the MAGs remain unconfirmed. The metabolism of methanethiol and DMS represents a major component of the global sulfur cycle (Carrión et al., 2017, 2019), indicating an additional role for the active soil microbiome on the metabolism of climactically relevant sulfur species. Screening of the MAGs also identified two MAGs identified as members of the *Candidatus* Patescibacteria, which contains the bacterial candidate phyla radiation. Analysis of these MAGs identified a minimal presence of metabolic pathways, reinforcing the streamlined nature of the genomes of these taxa (Lemos et al., 2019; Narasingarao et al., 2012; Tian et al., 2020). However, these MAGs did contain rRNA genes, which allowed their taxonomic identity to be better resolved to *Candidatus* Nomurabacteria (bin 35) and *Candidatus* Berkelbacteria (bin 21).

### Identification of viral diversity

Vibrant extracted 644 Viral MAGs from the methanol‐enriched soil metagenome (Supplementary Figure 2). RNA viruses may also be present and enriched in these samples, but these cannot be detected with the sequencing approach used in this study. Of these, 626 were low quality, 16 were medium quality and two were high quality, with 560 lytic and 84 lysogenic. Four of the low‐quality viral MAGs were identified as circular, with the rest all identified as linear. The two high‐quality MAGs were identified as lytic and did not cluster with any other phage in the RefSeq or NCBI databases, but screening of individual genes from the genome identified matches against the genomes of betaproteobacteria; this suggests that this may represent the host diversity of this virus. The most abundant pathways within the viral MAGs are related to amino acid, metabolite and cofactor synthesis (Supplementary Figure 3); these pathways are related to the enhanced replication of viral particles (Zhang et al., 2021). The detection of this high number of viral MAGs in a metagenome generated from the ^13^C‐labelled DNA from an SIP experiment would indicate that these phages were either actively infecting and lysing cells at the termination of the experiment or were lysogenic and contained in the genomes of actively replicating microbes. The high abundance of lytic phages also supports that this enhanced viral activity was in response to the increased abundance of specific taxa within the soil. Phages have previously been identified in SIP experiments investigating carbon cycling in soil (Barnett & Buckley, 2023; Greenlon et al., 2022; Haig et al., 2015; Lee et al., 2012), with labelling identified across a variety of ^13^C enrichment regimes (e.g., labelled plant tissue and ^13^CO_2_ fed plant rhizo‐exudates); this ability to delineate the active phage population from preserved environmental DNA represents a powerful technique to investigate their population dynamics and the resultant impact on carbon cycling.

## CONCLUSION

The generation of focussed metagenomes *via* SIP experiments represents a powerful means to identify the active taxa performing a specific metabolic group within an environment. Whilst cross‐feeding consistently occurs across SIP experiments, the ability to identify the genomic contexts of labelled taxa enhances the means through which primary utilizers can be identified. Furthermore, the characterisation of MAGs involved in the secondary utilisation of this carbon represents an approach to further elucidate the biogeochemical cycling and genetic diversity of uncultivated taxa.

## AUTHOR CONTRIBUTIONS

**Michael C. Macey:** Data curation (equal); investigation (equal); methodology (equal); writing – original draft (equal); writing – review and editing (equal).

## CONFLICT OF INTEREST STATEMENT

The authors declare no conflicts of interest.

## Supporting information


**Supplementary Figure 1.** Taxonomic identity of MAGs generated from the metagenomes generated from DNA extracted ^13^C methanol enriched soil.

**Supplementary Figure 2.** Principal component analysis of viral assemblies assembled from metagenomes generated from DNA extracted ^13^C methanol enriched soil.

**Supplementary Figure 3.** Abundance and identity of auxiliary metabolic genes identified within binned viral sequence data generated from the methanol‐enriched soil metagenomes with the program VIBRANT.

## Data Availability

The original metagenome data are available in GenBank under BioProject number PRJNA533040.
