# Supplementary figures and images for: Genome‐resolved metagenomics identifies novel active microbes in biogeochemical cycling within methanol‐enriched soil
Source: Environ Microbiol Rep. 2024 Apr 4;16(2):e13246. doi: 10.1111/1758-2229.13246 (PMC10994693; doi:10.1111/1758-2229.13246)

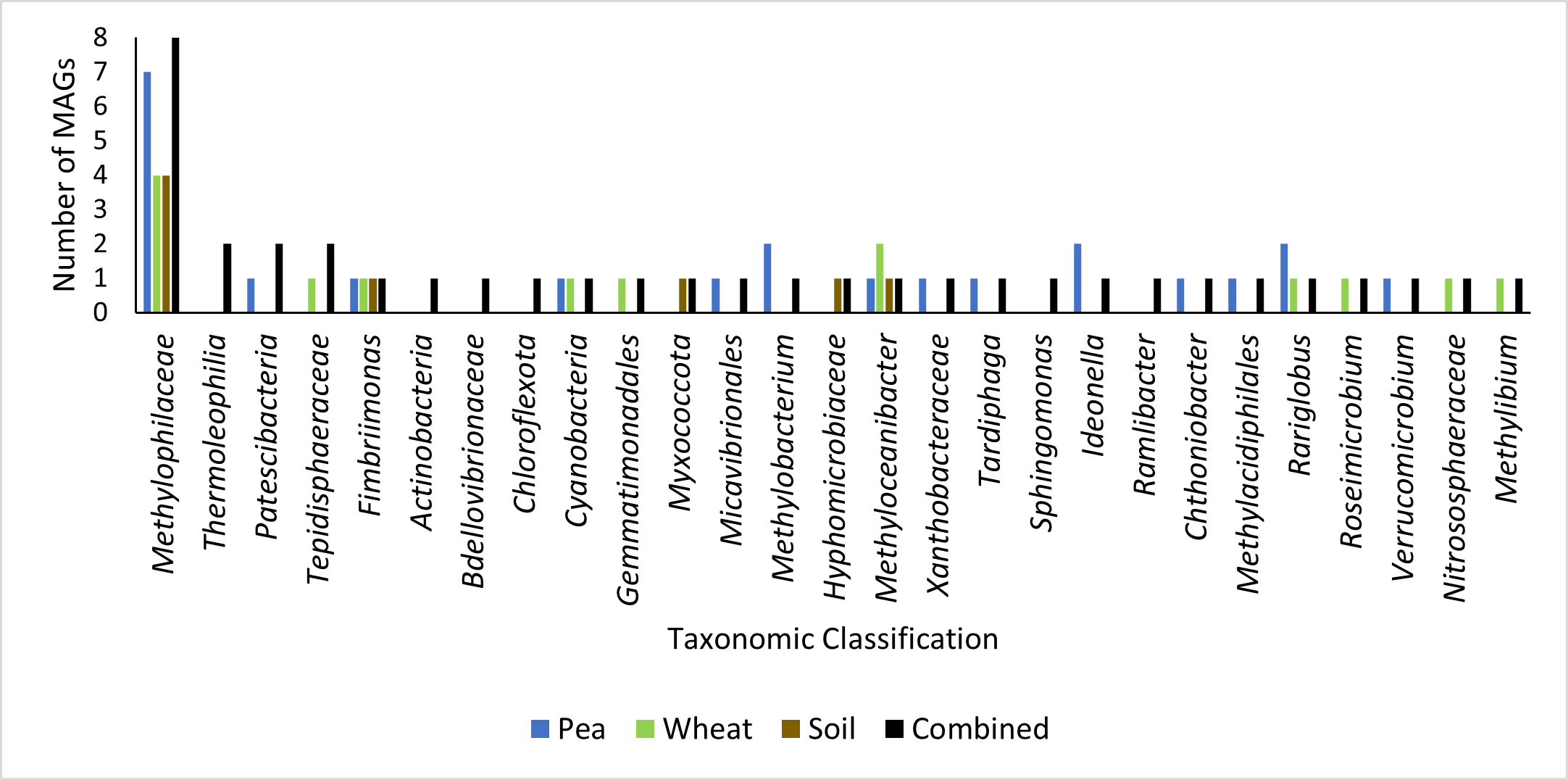

Supplement: Supplementary file 1 — Supplementary Figure 1. Taxonomic identity of MAGs generated from the metagenomes generated from DNA extracted 13C methanol enriched soil. [file EMI4-16-e13246-s002.png]

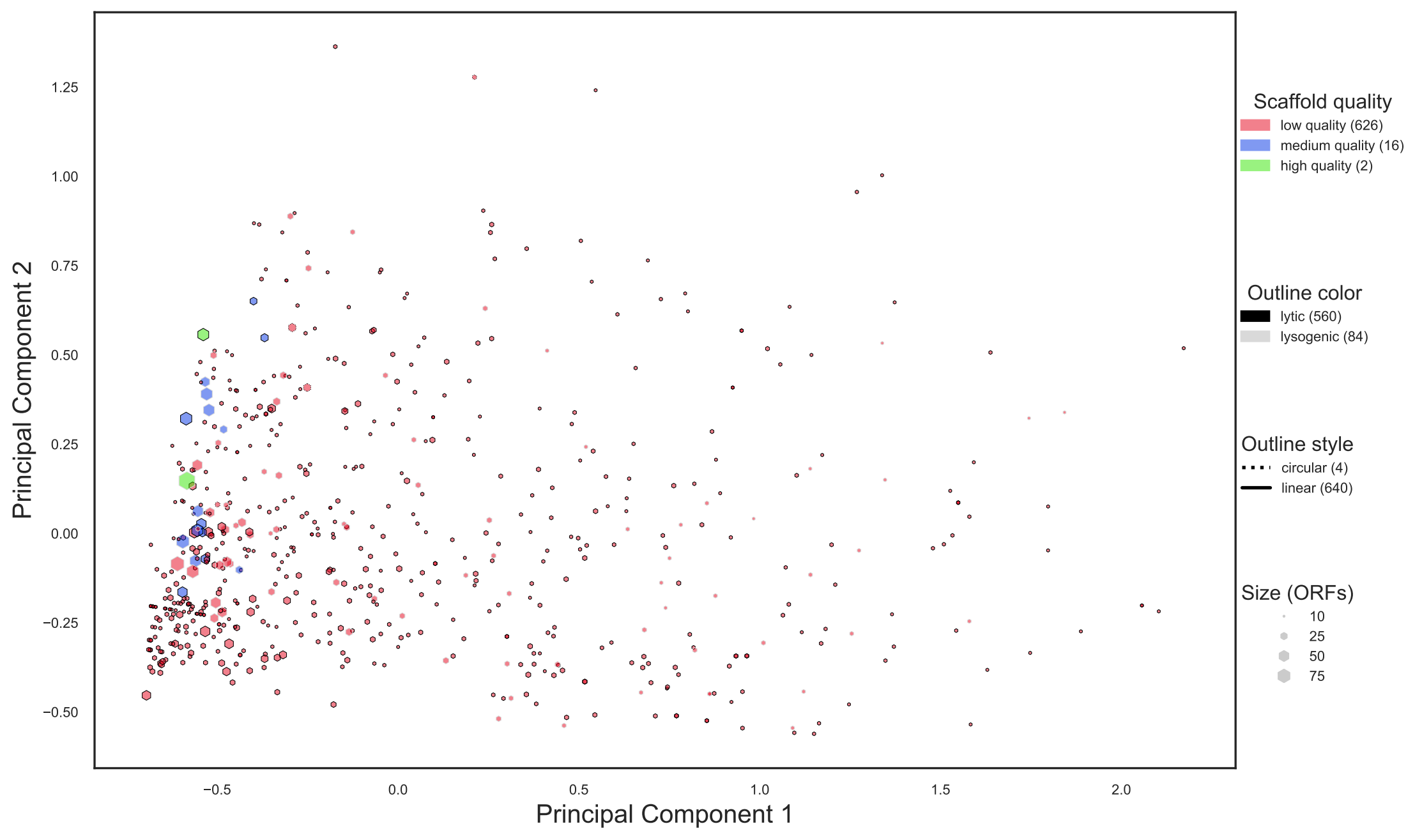

Supplement: Supplementary file 2 — Supplementary Figure 2. Principal component analysis of viral assemblies assembled from metagenomes generated from DNA extracted 13C methanol enriched soil. [file EMI4-16-e13246-s003.pdf]

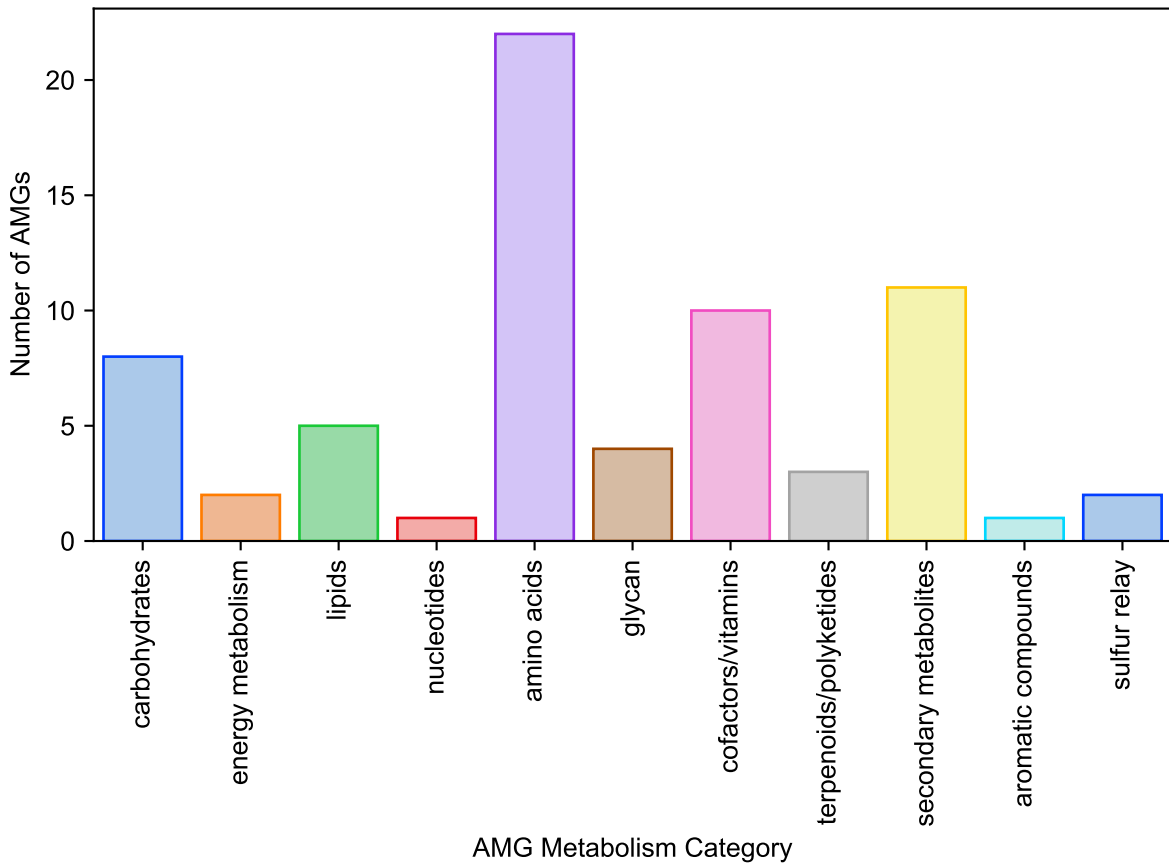

Supplement: Supplementary file 3 — Supplementary Figure 3. Abundance and identity of auxiliary metabolic genes identified within binned viral sequence data generated from the methanol‐enriched soil metagenomes with the program VIBRANT. [file EMI4-16-e13246-s001.pdf]
